# Osteopontin Blockade Immunotherapy Increases Cytotoxic T Lymphocyte Lytic Activity and Suppresses Colon Tumor Progression

**DOI:** 10.3390/cancers13051006

**Published:** 2021-02-28

**Authors:** John D. Klement, Dakota B. Poschel, Chunwan Lu, Alyssa D. Merting, Dafeng Yang, Priscilla S. Redd, Kebin Liu

**Affiliations:** 1 Department of Biochemistry and Molecular Biology, Medical College of Georgia, Augusta, GA 30912, USA; Jklement@augusta.edu (J.D.K.); Dakbooth@augusta.edu (D.B.P.); Amerting@augusta.edu (A.D.M.); Dyang@augusta.edu (D.Y.); Predd@augusta.edu (P.S.R.); 2 Georgia Cancer Center, Augusta University, Augusta, GA 30912, USA; 3 Charlie Norwood VA Medical Center, Augusta, GA 30904, USA; 4 School of Life Sciences, Tianjin University, Tianjin 300072, China; Clu@augusta.edu; 5 Chemedimmune Inc., Augusta, GA 30912, USA

**Keywords:** osteopontin, immune checkpoint, cytotoxic T lymphocytes, MSS, OPN neutralization, PD-L1

## Abstract

**Simple Summary:**

Despite the breakthrough in human cancer immunotherapy, colorectal cancer, except for the small subset of microsatellite instable colorectal cancer (MSI, ~4% total cases), is one of the few human cancers that does not respond to current immune checkpoint inhibitor (ICI) immunotherapy. CTLs are present in both MSI and microsatellite stable (MSS) human colon carcinoma, suggesting that PD-L1-independent mechanisms may exist and suppress CTL activation in the colon tumor microenvironment. We determined that osteopontin (OPN) inhibits tumor-specific cytotoxic T lymphocyte (CTL) lytic activity to promote colon tumor growth in vivo. Accordingly, OPN blockade immunotherapy using OPN neutralization monoclonal antibodies 100D3 and 103D6 suppressed colon tumor growth in vivo. Our findings indicate that 100D3 and 103D6 has the potential to be further developed for colorectal cancer immunotherapy.

**Abstract:**

Human colorectal cancers are mostly microsatellite-stable with no response to anti-PD-1 blockade immunotherapy, necessitating the development of a new immunotherapy. Osteopontin (OPN) is elevated in human colorectal cancer and may function as an immune checkpoint. We aimed at elucidating the mechanism of action of OPN and determining the efficacy of OPN blockade immunotherapy in suppression of colon cancer. We report here that OPN is primarily expressed in tumor cells, myeloid cells, and innate lymphoid cells in human colorectal carcinoma. Spp1 knock out mice exhibit a high incidence and fast growth rate of carcinogen-induced tumors. Knocking out Spp1 in colon tumor cells increased tumor-specific CTL cytotoxicity in vitro and resulted in decreased tumor growth in vivo. The OPN protein level is elevated in the peripheral blood of tumor-bearing mice. We developed four OPN neutralization monoclonal antibodies based on their efficacy in blocking OPN inhibition of T cell activation. OPN clones 100D3 and 103D6 increased the efficacy of tumor-specific CTLs in killing colon tumor cells in vitro and suppressed colon tumor growth in tumor-bearing mice in vivo. Our data indicate that OPN blockade immunotherapy with 100D3 and 103D6 has great potential to be further developed for colorectal cancer immunotherapy and for rendering a colorectal cancer response to anti-PD-1 immunotherapy.

## 1. Introduction

Human colorectal cancer is a type of highly immunogenic tumor [1,2], but only the small subset of microsatellite instable (MSI) colorectal cancer, which accounts for only approximately 4% of human colorectal cancer cases, responds to anti-PD-1 immunotherapy [3]. High tumor mutation burdens (TMB) may serve as neoantigens to generate tumor-reactive cytotoxic T lymphocytes (CTLs) in MSI colorectal cancer [3,4]. However, CTL infiltrates are present in both MSI and microsatellite stable (MSS) human colon carcinoma [5], suggesting that other immune checkpoints may compensate for PD-L1 function in suppression of tumor-infiltrating CTL effector function in human colorectal carcinoma [4].

Osteopontin (OPN), a phosphorylated glycoprotein, was first identified as a secreted protein in bone and later discovered as an intracellular protein [6]. It has since been determined that OPN is often overexpressed in cells of the tumor microenvironment to promote tumor growth and progression in human cancer patients [7,8,9,10]. OPN protein is elevated in the peripheral blood of human cancer patients and OPN overproduction is associated with worse prognosis in human cancers [10,11,12,13,14,15]. OPN may directly regulate tumor cell proliferation, migration through binding to CD44 or the integrin receptors on tumor cell surface [16,17,18,19]. Tumor-secreted OPN also binds to α_V_β_3_ integrin and CD44 on fibroblasts to reprogram normal fibroblasts into tumor-promoting cancer-associated fibroblasts in mammary carcinoma [20]. OPN also modulates immune cell function in various disease settings [16,21]. In fact, OPN was also initially identified as a regulator of T cell activation and was termed the early T cell activation gene (Eta-1) [22]. Early studies have determined that OPN regulates type-1 immunity to viral and bacterial infection through regulation of IL-12 and IL-10 expression in myeloid cells and T cells [23,24]. In addition, OPN-deficient mice exhibit altered invariant NKT (iNKT) cell maturation and function with downregulation of the iNKT cell receptor, reduced IL-4 production and decreased Fas ligand expression, leading to reduced Fas/FasL-dependent cytotoxicity against hepatocytes [25]. 

Emerging experimental data indicate that, unlike its functions as an immune activator under physiological conditions, OPN functions as an immune suppressor in the tumor microenvironment through regulating myeloid cells and T cells [9,26,27,28,29]. OPN induces M2 macrophage polarization, maintains M2 macrophage phenotypes, and acts as a chemoattractant for tumor-associated macrophages [26,30,31]. In return, tumor-associated macrophages produce abundant OPN that not only directly targets tumor for migration and progression [32,33], but also suppresses T cell activation and function [26,27,34,35]. These findings suggest that OPN may function as an immune checkpoint in T cells in the tumor microenvironment, which underlies colorectal tumor immune evasion and non-response to anti-PD-1 immunotherapy [26,27,36]. We aimed at testing this hypothesis by generating OPN neutralization monoclonal antibodies to block OPN and T cell interactions. Our data indicate that OPN blockade immunotherapy increases tumor-specific CTL effector function and decreases colon tumor growth in vivo.

## 2. Materials and Methods

### 2.1. Human Colorectal Carcinoma Specimens 

Human colorectal carcinoma and matched adjacent non-neoplastic colon were provided by Cooperative Human Tissue Network Southern Division (CHTN, Duke University Medical Center) (Table S1). 

### 2.2. Patient Dataset Analysis

OPN mRNA level and survival datasets were extracted from the TCGA and ONCOLNC databases. OPN mRNA expression level between tumor tissues and the respective normal tissues was plotted. Kaplan–Meier survival curves were generated at a 50:50 expression high and low cut off. Single cell RNA sequencing (scRNA-Seq) of human colon and breast cancer raw datasets were extracted from the GEO database (GSE146771 and GSE114727) [37,38]. The datasets were analyzed using R package.

### 2.3. Mice

Balb/c, C57BL/6, and *Spp1* KO mice were purchased from Jackson Laboratory (Bar Harbor, ME). Both male and female mice were used. All mice used in this study were 2–3 months old at the start of the experiment. 

### 2.4. Methylcholanthrene (MCA) Induction of Tumor Development

The chemical MCA is a highly carcinogenic polycyclic aromatic hydrocarbon and induces immunogenic fibrosarcoma [39,40]. MCA was dissolved in peanut oil and injected to the right flank of mice (100 g/100 L). A single tumor nodule forms approximately 2–3 months later at the site of MCA injection. Mice were monitored for tumor growth.

### 2.5. Colon Tumor Mouse Model 

CT26 cells were injected to mice (2 × 10^5^ cells/mouse) through the lateral tail vein. For quantification experiments, mice were sacrificed at the experimental endpoint and tumor nodules were quantified as described [41].

### 2.6. OPN Neutralization Monoclonal Antibody Generation

Five C57BL/6 mice were immunized with 50 g/mouse recombinant OPN protein (Biolegend, San Diego, CA, USA). The immunized mice were boosted with 25 g OPN protein/mouse at days 14, 28, 35, and 50 after initial immunization. Mice serum was tested 7 days after each boost for immune response to OPN protein by ELISA assay. The three mice with the highest OPN antibody titer were selected for spleen cell electro-fusion with SP2/0 myeloma cells. Fused cells from each cell fusion was plated into 96-well plates. A total of 90 plates were screened for binders by ELISA with OPN protein. The top 126 parental clones were screened by ELISA for binding to OPN and by T cell proliferation rescue assay in anti-CD3/CD28/OPN-coated plates. The top four positive primary clones were subcloned by limited dilution to generate four single cell clones (89G9, 100D3, 100G2, and 103D6). These four monoclonal antibodies are deposited in Bio X Cells and purified in Bio X Cells to low endotoxin.

### 2.7. Cell Lines

The mouse colon carcinoma CT26 cell line and mammary carcinoma 4T1 cell line were obtained from American Type Culture Collection (ATCC) (Manassas, VA). ATCC characterized these cells by morphology, immunology, DNA fingerprint, and cytogenetics. To create the OPN Ko cell line, HEK293FT cells were co-transfected with pCMV-VSV-G (#8454, Addgene, Watertown, MA, USA), psPAX2 (#12260, Addgene, Watertown, MA, USA) and lentiCRISPRv2 (Genscript, Piscataway, NJ, USA) plasmids using Lipofectamine 2000 (Life Technologies, Carlsbad, CA, USA). Scramble and OPN sgRNA sequences are 5′-CTCGTATCTTTTCCCACGGC-3′, and 5′-AAGGTGAAAGTGACTGATTC-3′, respectively. After forty-eight hours, lentiviral particles were harvested and cell lines were transduced with polybrene. Seventy-two hours post-transduction, cells were harvested and puromycin-selected (5 µg/mL) for three days. Cell phenotype was confirmed by measuring culture supernatant OPN protein level.

### 2.8. Co-Culture System

Unless otherwise stated, CT26 cells were seeded 1 × 10^5^/well into a 96-well U-bottom plate. The gp70 antigen-specific T-cell line 2/20 was added at 0:1, 1:16, 1:8, 1:4, 1:2, and 1:1 ratios (E:T). For the OPN mAb blocking assay, tumor cells were cultured for 24 h, OPN neutralization mAbs and IgG were added and cultured overnight. Floating and adherent cells were collected, stained, and analyzed by flow cytometry.

### 2.9. T Cell Proliferation Assay

CD3^+^ T cells were purified from BALB/c mouse spleen cells with the MojoSort mouse CD3 T cell isolation kit (Biolegend, San Diego, CA, USA) according to the manufacturer’s instructions. For T cell proliferation assay, a 96-well culture plate was coated with anti-mouse CD3 (8 g/mL), anti-mouse CD28 MAbs (10 g/mL), and recombinant protein overnight. The purified T cells were labeled with CFSE (Life Technologies, Carlsbad, CA, USA) and then seeded in the plate at a density of 1.5 × 10^5^ cells/well in 150 μL medium for 3 days. Cells were analyzed by flow cytometry.

### 2.10. Immunohistochemistry

The formalin fixed paraffin-embedded tissue sections were stained with anti-human OPN antibody (R and D System, Minneapolis, MN, USA) as previously described [5].

### 2.11. OPN ELISA and OPN Antibody Binding to OPN Protein Analysis

Cell culture supernatant and mouse serum were analyzed for OPN protein concentration using the mouse OPN ELISA kit (R and D System, Minneapolis, MN) according to the manufacturer’s instructions. To determine antibody binding affinity. Recombinant OPN protein was coated at 1 μg/mL in 100 μL PBS/well overnight. The wells were washed, blocked, and then hybridoma culture supernatant was added to the coated wells at various dilutions. Peroxidase-AffiniPure Goat Anti-Mouse IgG was used as secondary antibody and the assay was performed using the ELISA kit (Biolegend, San Diego, CA, USA) according to the manufacturer’s instructions. 

### 2.12. Flow Cytometry

General flow cytometry staining protocol is as follows. Samples were incubated at 4 °C for 30 min. Samples were then washed with PBS, fixed in 2% paraformaldehyde, and acquired on a FACSCalibur with CellQuestPro or LSRFortessa with BD Diva 8.01 (BD Biosciences, San Jose, CA, USA). All flow cytometry data analysis was conducted with FlowJo v10.6.0 (BD Biosciences, San Jose, CA, USA). Annexin V-APC was obtained from Biolegend.

### 2.13. Statistical Analysis

Unless otherwise indicated, all statistical analysis was conducted using Prism8 (Graphpad, San Diego, CA, USA) and *p*-values were calculated by a two-tailed Student’s *t*-test. Significance between survival groups was computed by two-sided log-rank test. 

## 3. Results

### 3.1. OPN Expression Is Elevated in Human Cancers

**Figure 1:**
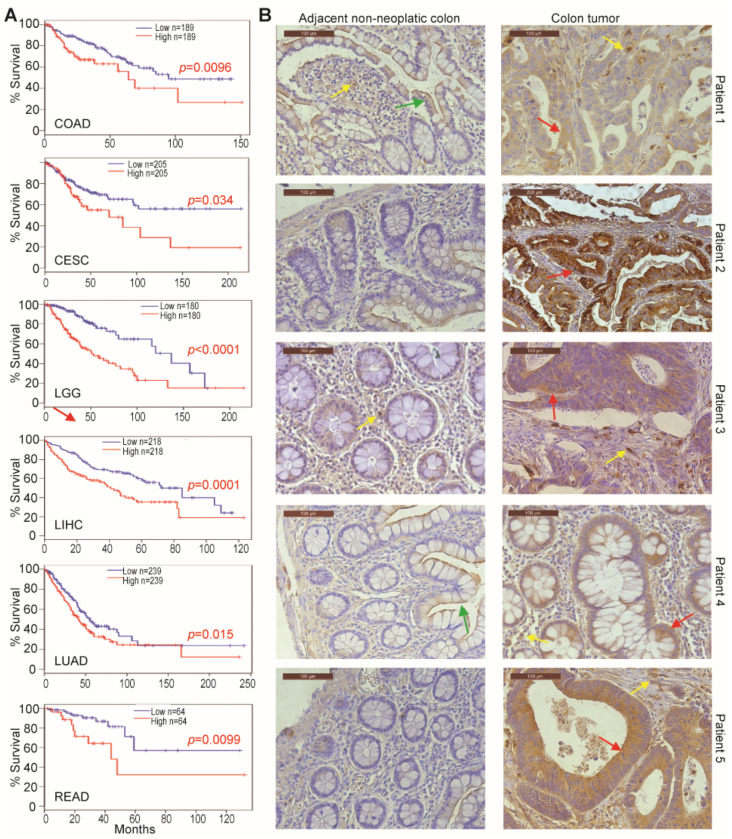
OPN protein level in non-neoplastic colon and colon carcinoma. (**A**) OPN mRNA expression level and survival datasets were extracted and plotted for survival. CESC: Cervical squamous cell carcinoma and endocervical adenocarcinoma; COAD: Colon adenocarcinoma; LGG: Brain lower grade glioma; LIHC: Liver hepatocellular carcinoma; LUAD: Lung adenocarcinoma; READ: Rectum adenocarcinoma. (**B**) Human colon carcinoma (*n* = 5) and matched non-neoplastic colon (*n* = 5) were stained with OPN-specific antibody. Green arrows indicate normal colon epithelium. Yellow arrows indicate myeloid cells. Red arrows indicate carcinoma cells. Shown are representative images. Scale bar = 100 μM.

OPN expression datasets were compared between tumor tissues and the respective normal tissues in human cancer patients. The OPN expression level was significantly higher in tumor tissues than in normal tissues in twenty-eight of the thirty human cancers analyzed (Figure S1). Analysis of correlation between OPN mRNA level and patient survival data revealed that the OPN mRNA expression level is inversely correlated with patient survival time in six cancers including colon and rectum cancer (Figure 1A). Immunohistochemical analysis of human colorectal tumor and matched adjacent non-neoplastic colon tissues indicates the OPN protein level is dramatic higher in the tumor tissues than in the matched non-neoplastic colon in all five colorectal cancer patients (Figure 1B). 

**Figure 2:**
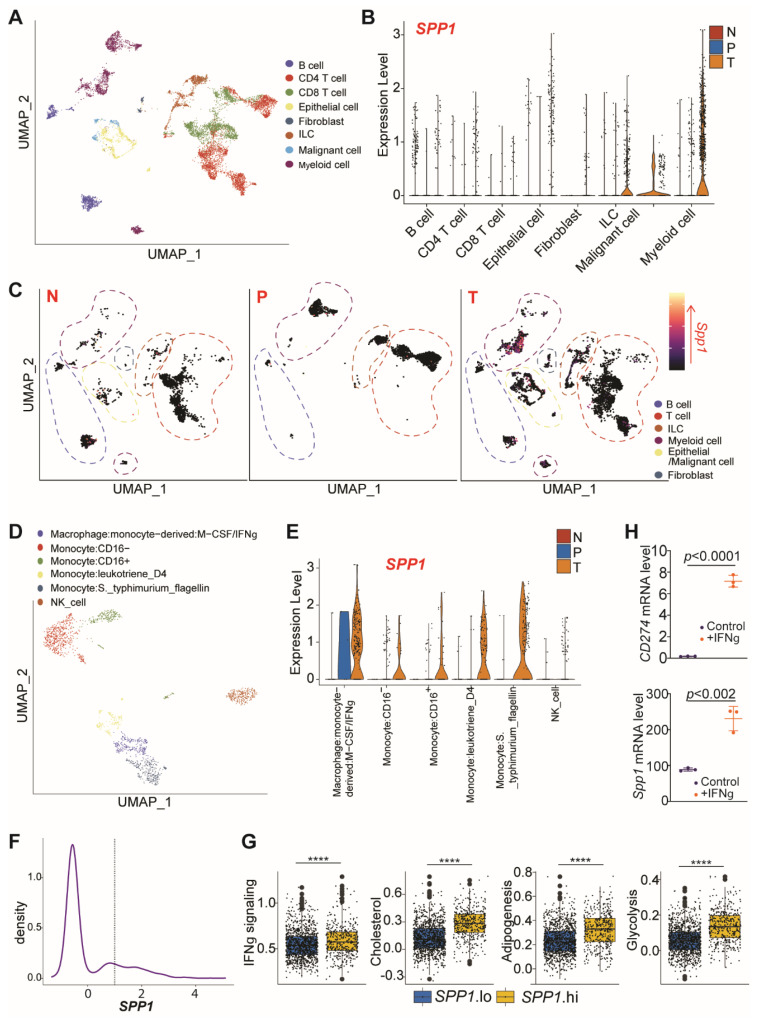
OPN expression profiles in the single cell level in human colorectal cancer patients. (**A**) UMAP projection of GSE146771 CRC 10× scRNA-seq data. Cells are annotated according to author’s designation. (**B**) Expression of *SPP1* in indicated cells, sub-grouped by tissue of origin. N: Normal Colon; P: PBMC; T: CRC. (**C**) UMAP projection of *SPP1* expression, with cells sub-grouped by tissue of origin. (**D**) Sub-grouping and re-clustering of author-designated myeloid cells. Cluster identities were determined by comparison to Human Protein Atlas reference samples by SingleR. (**E**) Expression of *SPP1* in indicated cell clusters, sub-grouped by tissue of origin. (**F**) Density plot of *SPP1* expression across all myeloid cells. Cells with expression level of *SPP1* > 1 were designated as *SPP1*.hi; the remainder designated as *SPP1*.lo. (**G**) Comparison of hallmark gene signature scores computed by VISION between *SPP1*.hi and *SPP1*.lo cells. **** *p* < 1 × 10^−15^. (**H**) CT26 cells were treated with IFNγ for approximately 24 h, and analyzed by qPCR for Cd274 and Spp1 expression. β-actin was used as internal control.

To investigate the cellular source of OPN in colorectal cancer patients, we mined scRNA-Seq raw datasets deposited in the GEO database (GSE146771) [37]. Colorectal tumor-resident immune cells were annotated (Figure 2A). Cellular subtype analysis demonstrated that principally myeloid cells, as well as innate lymphoid cells (ILCs) and malignant cells, drove increased OPN levels (Figure 2B). Expression of *SPP1* was enriched in colorectal tumor tissues compared to matched PBMC and healthy colon (Figure 2C). We then sought to elucidate whether *SPP1* expression was driven by expansion of a subset of myeloid cells, such as myeloid-derived suppressor cells, with high expression of *SPP1* at baseline, or was due to a tumor-resident state. To do so, myeloid cells were re-extracted and re-clustered. Cluster identity was determined by SingleR, a nonbiased, automatic cell-type annotator, by comparison to HPA reference transcriptomic data (Figure 2D) [42]. Elevation of *SPP1* expression was globalized across myeloid cell states, with all sub-clusters showing increased *SPP1* levels in colorectal tumor relative to normal colon, suggesting that tumor residency is sufficient for inducing *SPP1* expression across the myeloid lineage (Figure 2E). To examine the cell-type associated with enhanced *SPP1*, myeloid cells were sub-grouped into *SPP1*-high and low populations and scored for GSEA hallmark pathway enrichment. Comparative analysis across these cells showed that *SPP1*-high cells demonstrated enhanced transcription of genes associated with IFNγ signaling, cholesterol metabolism, adipogenesis, and glycolysis (Figure 2F,G).

Based on the scRNAseq results correlating OPN expression with IFNγ signaling pathways, we hypothesized that OPN may contribute to ‘adaptive immune resistance’ as PD-L1, in which tumor-immune interactions induce a counterregulatory program that protects tumor cells from immune clearance. In this model, tumor PD-L1, among other proteins, is upregulated by CTL-derived IFNγ, which is thought to prevent CTL-mediated killing. To test this hypothesis, we treated CT26 cells with IFNγ in vitro. As expected, CD274 expression was robustly induced following IFNγ treatment. Supporting our hypothesis, SPP1 was similarly increased (Figure 2H).

**Figure 3:**
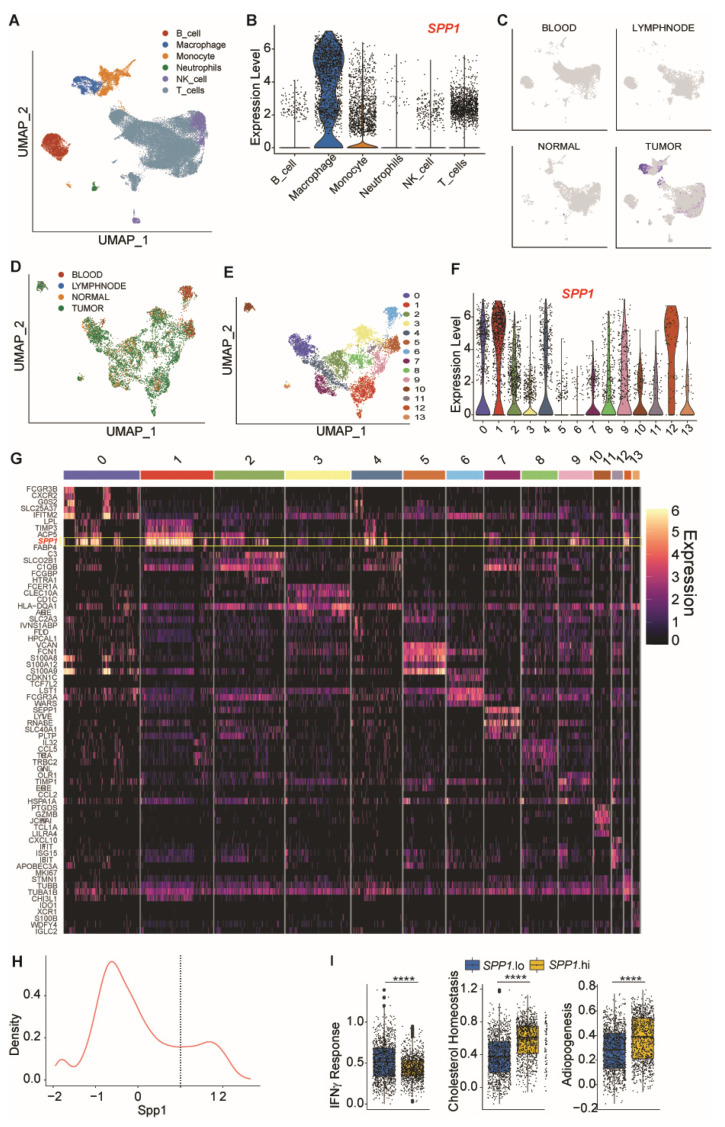
Analysis of OPN expression profiles in cells of the human breast tumor microenvironment in single-cell level. Datasets were extracted from the human breast tumor single-cell RNA-Seq datasets (GEO accession: GSE114727) and analyzed using R package. (**A**) UMAP projection of GSE114725 scRNA-seq data. Cluster identities were determined by comparison to Human Protein Atlas reference samples by SingleR. (**B**) Expression of SPP1 in indicated cell clusters. (**C**) UMAP projection of SP1 expression, with cells sub-grouped by tissue of origin. (**D**) Sub-grouping and re-clustering of myeloid (macrophage, monocyte and neutrophil) cell populations. Cells labeled by tissue of origin. (**E**) UMAP projection of myeloid cells, labeled by k-means cluster identity. (**F**) SPP1 expression in indicated cell clusters. (**G**) Heatmap of top 5 differentially expressed markers by each cluster, as determined by Seurat’s “FindAllMarkers” function. (**H**) Density plot of SPP1 expression across all cells identified as macrophages. Cells with expression level of SPP1 > 1 were designated as SPP1.hi; the remainder designated as SPP1.lo. (**I**) Comparison of hallmark gene signature scores computed by VISION between SPP1.hi and SPP1.lo cells. **** *p* < 1 × 10^−15^.

Analysis of scRNA-Seq dataset from human breast cancer patients (GSE114727) [38] validated cellular OPN expression profiles in tumor-resident cells (Figure 3A–E). As in colorectal cancer, elevation of SPP1 expression was localized to and globalized across myeloid cell states, with all sub-clusters showing increased SPP1 levels (Figure 3F–H). Comparative analysis across SPP1-hi and SPP1-lo cells showed that SPP1-high myeloid cells also demonstrated modulation of IFN signaling, cholesterol metabolism, and adipogenesis (Figure 3H,I).

### 3.2. OPN Promotes Tumorigenesis and Tumor Development

**Figure 4:**
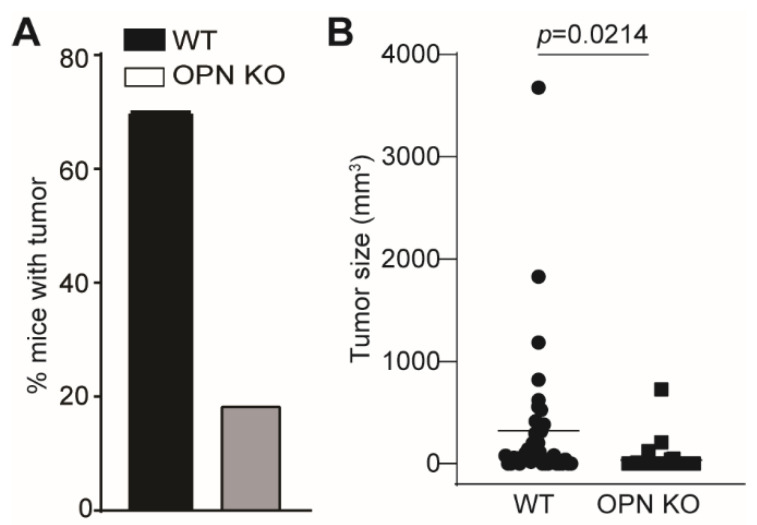
OPN promotes tumor development. (**A**) WT (*n* = 37) and *Spp1* KO (*n* = 32) mice were injected with MCA and monitored for tumor development. Shown are percentages of WT and OPN KO mice with tumor 102 days after MCA injection. (**B**) The tumor sizes were measured in WT and OPN KO mice and plotted.

To functionally validate the above findings, we used carcinogen MCA to induce sarcoma in WT and OPN KO mice. MCA induces a single tumor nodule in the site of injection. Tumor formed in 70.27% (26/37) WT mice and 18.75% (6/32) OPN KO mice (Figure 4A). Analysis of tumor size indicates that OPN deficiency results in a significant decrease in the overall tumor growth rate in tumor-bearing mice (Figure 4B). These findings validate that OPN promotes tumor initiation and growth in vivo.

### 3.3. OPN Inhibits T Cell Activation to Suppress CTL Cytotoxicity and Promote Tumor Growth

**Figure 5:**
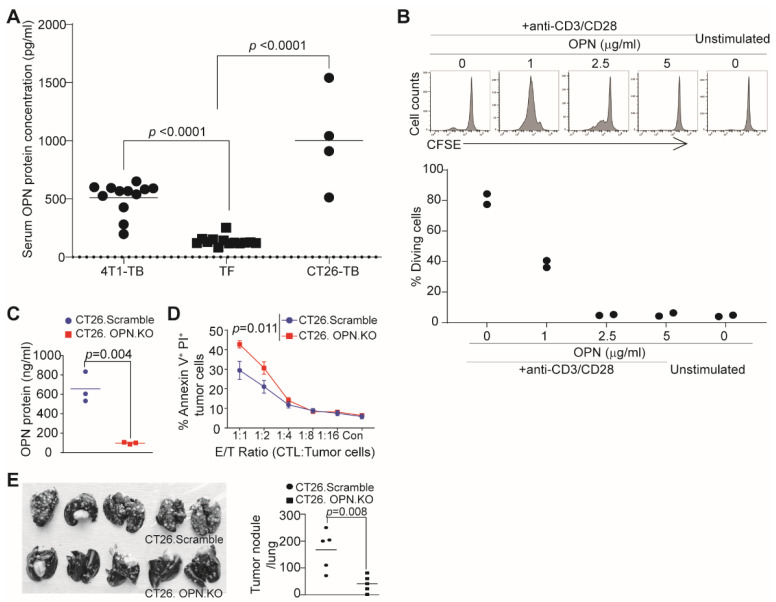
OPN inhibits tumor-specific CTL lytic efficacy and promotes colon tumor growth. (**A**) Serum specimens collected from tumor-free, 4T1 tumor-bearing, and CT26 tumor-bearing mice, and measured for OPN protein level by ELISA. (**B**) CFSE-labelled CD3^+^ T cells were cultured in anti-CD3 (8 g/mL) and CD28 (10 g/mL)-coated plates in the presence of the indicated coated OPN protein concentrations for 3 days. Cells were analyzed by flow cytometry. Shown is histograph of CFSE intensity (top panel) and quantification of T cell division (bottom panel). (**C**) CT26.Scramble and CT26.OPN KO cells were cultured for 24 h. Supernatants were collected and measured for OPN protein level. (**D**) CT26.Scramble and CT26.OPN KO cells were co-cultured with the tumor-specific CTLs for 24 h. Both floating and adherent cells were collected, stained with Annexin V and PI, and analyzed by flow cytometry. (**E**) CT26.Scramble and CT26.OPN KO cells (1 × 10^6^ cells/mouse) were injected to BALB/c mice intravenously. Lungs were examined 14 days later for tumor nodules (left panel) and quantification of tumor nodules.

OPN is a secreted protein that is elevated in human cancer patient peripheral blood [10,13,15,29,41]. Analysis of tumor-bearing mice validated that OPN is elevated in the serum of tumor-bearing mice (Figure 5A). Furthermore, OPN exhibits potent inhibitory activity against T cell activation and proliferation (Figure 5B). To determine the function of tumor-expressed OPN in tumor–T cell interaction, we knocked out *Spp1* in CT26 cells (Figure 5C). Analysis of CTL effector function in a tumor-CTL co-culture model determined that knocking out OPN in the target tumor cells results in a significant increase in efficacy of the tumor-specific CTL in killing the target tumor cells (Figure 5D). To determine whether this finding can be translated to tumor suppression in vivo, CT26.scramble and CT26 OPN KO tumor cells were injected to mice. Significantly more tumor nodules were formed in the WT mice than in the OPN KO mice (Figure 5E). 

### 3.4. OPN Neutralization Increases CTL Lytic Activity

**Figure 6:**
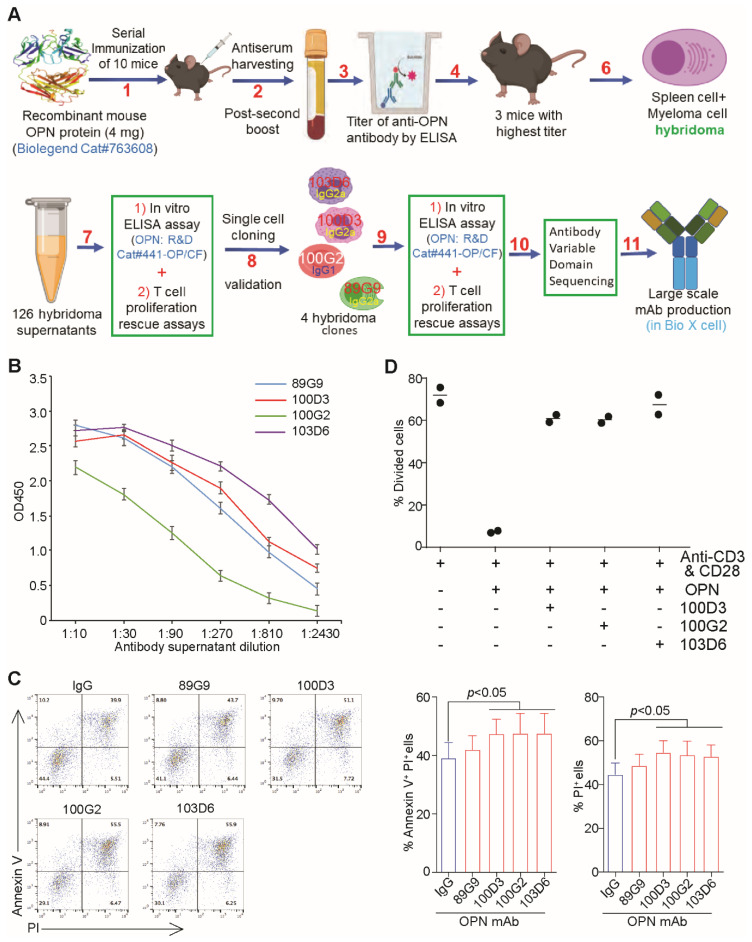
OPN neutralization mAbs increases CTL tumor-lysing efficacy. (**A**) Scheme of OPN neutralization monoclonal antibody production as described in Materials and Methods. (**B**) The four OPN mAb clones were tested for binding affinity to OPN protein by ELISA. (**C**) CT26 cells were seeded in 96-well U bottom plates for 24 h. IgG and the indicated OPN mAbs and CTLs were added to the tumor cell culture for 24 h. Cell mixtures were collected, stained with CD8, Annexin V, and PI, and analyzed by flow cytometry. Representative dot plots are shown at the left, and tumor cell death was quantified and presented at the right. (**D**) CSFE-labelled CD3^+^ T cells were cultured in 96-well plates coated with anti-CD3, anti-CD28, and OPN as indicated. Cells were analyzed for CSFE intensity 3 days later by flow cytometry. Shown are quantification of % divided cells as determined by CFSE intensity.

The literature and our above findings that OPN is expressed in various resident cells in the tumor microenvironment suggest that targeting serum OPN protein, rather than targeting a particular cell type, is potentially an effective approach to block OPN function in cancer patients, which provides a strong rationale to develop OPN neutralization monoclonal antibodies. We have therefore developed four OPN monoclonal antibodies for blocking OPN binding to T cells (Figure 6A). All four mAbs have higher OPN protein-binding affinity (Figure 6B). Three of the four mAb clones significantly increased the lytic activity of a tumor-specific CTL in lysing the target CT26 tumor cells in vitro (Figure 6C). Furthermore, these OPN mAbs are effective in reversing OPN-mediated suppression of T cell activation in vitro (Figure 6D). 

### 3.5. OPN Blockade Immunotherapy Suppresses Colon Tumor Growth In Vivo

**Figure 7:**
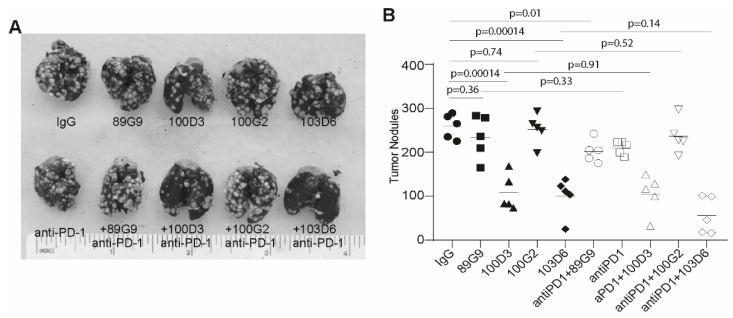
OPN blockade immunotherapy suppresses colon tumor growth in vivo. (**A**) CT26 cells were injected i.v. into tail veins of BALB/c mice. Three days later, IgG and the indicated OPN mAbs (200 μg/mouse) were injected to the tumor-bearing mice s.c. either alone or in combination with anti-PD-1 (200 μg/mouse) (*n* = 5) every 3 days. (**B**) Mice were sacrificed on day 14 and analyzed for lung tumor nodules. Shown are representative tumor-bearing lungs of each treatment group (top panel). The tumor nodule number was quantified and presented at the bottom panel.

To determine whether the above findings can be translated into colon tumor growth suppression in vivo, we injected CT26 tumor cells to mice. The tumor-bearing mice were then treated with OPN mAbs alone or in combination with anti-PD-1 mAb. OPN mAb clones 100D3 and 103D6 significantly suppressed CT26 tumor growth in mouse lungs (Figure 7A). However, 100D3 and 103D6 did not significantly increase the efficacy of anti-PD-1 immunotherapy (Figure 7B). Nevertheless, OPN mAb clones 100D3 and 103D6 are effective in enhancing colon tumor growth control in immune competent mice.

## 4. Discussion

In human cancer patients and tumor-bearing mice, OPN is produced by various resident cells in the tumor microenvironment [26,27,32,37,38]. Targeting OPN-producing cells is therefore challenging. OPN is secreted to the peripheral blood in cancer patients [10,13,15,29,43]. An OPN neutralization mAb is thus potentially an effective agent to block OPN function in both tumor promotion and in immune suppression [9,13,31,32,44]. OPN neutralization antibodies were initially developed for inflammation-mediated diseases, including osteoporosis, hepatitis, arthritis, and osteoporosis [16,45,46]. The OPN monoclonal antibody 23C3 treatment decreased bone loss associated with oophorectomies [45]. 23C3 was found to be effective in reducing pro-inflammatory cytokines and promoted the apoptosis of type-II collagen activated T cells to suppress osteoporosis [47]. Another OPN neutralization mAb, C2K1, was able to decrease collagen-induced arthritis in monkeys [46]. These studies demonstrated the efficacy of OPN neutralization mAbs in blocking OPN function in pro-inflammatory diseases primarily through targeting inflammatory cells [47,48]. However, these antibodies targets OPN interactions with inflammatory cells to suppress activation of inflammatory cells [46,47].

In human cancer patients and tumor-bearing mice, OPN receptors are diverse and expressed on tumor cells, T cells, and myeloid cells, and OPN uses its different domains to interact with different receptors [26,27,29]. Targeting OPN interactions with different cells (e.g., tumor cells, T cells, macrophages) may require different OPN neutralization mAbs. AOM1 is an OPN neutralization mAb that blocks the integrin αvβ3 binding site and the thrombin cleavage site on OPN. AOM1 treatment was effective in suppressing αvβ3-expressing tumor cell migration to suppress metastasis but not primary tumor growth [49]. OPN mAb, which targets the SVVYGLR motif in OPN protein, was effective in suppression of adult T cell leukemia growth and progression [50]. Furthermore, OPN monoclonal antibody MPIIIB10 delayed colon tumor growth and enhanced B cell-based vaccine-induced tumor growth suppression through inhibition of tumor OPN-induced myeloporesis [29]. These findings demonstrated the efficacy of OPN blockade monoclonal antibodies in suppression of tumor growth and progression through blocking OPN interactions with tumor cells and myeloid cells in the tumor microenvironment.

OPN was originally identified as a regulator of T cell activation and was termed the early T cell activation gene (Eta-1) [22]. In fact, suppression of cellular IFNγ production has been used as a quality control tool for a commercial OPN product (Biolegend, San Diego, CA, USA). We have recently determined that OPN directly engages T cells to suppress T cell activation [27]. These findings provide a strong rationale to block the OPN interaction with T cells to suppress tumor immune evasion. We have now developed four OPN neutralization mAbs. Two of these OPN monoclonal antibodies, 100D3 and 103D6, are effective in blocking OPN-mediated inhibition of T cell activation and CTL anti-tumor effector function. These two mAbs also show efficacy in suppression of colon tumor growth in immune-competent mice. 100D3 and 103D6 are thus potentially effective OPN neutralization antibodies for blocking OPN function in T cell suppression to suppress colon tumor immune evasion. 100D3 also has high affinity for human OPN protein. Humanization of 100D3 is currently under development.

Our analysis of mRNA level showed that OPN is primarily expressed in tumor cells, various subsets of myeloid cells and ILCs in human colorectal and breast cancer patients. The relative contributions of these cell types to the secreted OPN proteins in the patient peripheral remains to be determined. In addition, the relative contributions of these cell types in suppression of CTL recruitment and effector function in the tumor microenvironment also requires further study [26,27,36,51].

CTLs are present in both MSI and MSS human colorectal carcinoma [5], but the MSS human colorectal cancer does not respond to anti-PD-1 immunotherapy [3,4,52,53]. OPN may compensate PD-L1 function and thus renders human colorectal tumor cell resistance to anti-PD-1 immunotherapy. Therefore, blocking OPN may be an effective approach to increase anti-PD-1 efficacy in colon cancer immunotherapy. However, although we observed that 100D3 and 103D6 increased the efficacy of anti-PD-1 immunotherapy in suppression of colon tumor growth, the increase is not statistically significant. There are multiple OPN variant proteins [16,54,55]. In addition, as discussed above, there are several OPN receptors and different OPN domains bind to different OPN receptors [16,17,26,34,36,50,51,56,57]. Further studies are therefore needed to determine the efficacy of 100D3 and 103D6 in blocking OPN variants and different OPN–receptor interactions in the context of anti-PD-1 immunotherapy in suppression of colon tumor growth. 

## 5. Conclusions

Human colorectal cancers are mostly microsatellite-stable with no response to anti-PD-1 immunotherapy, necessitating the development of a new immunotherapy. Osteopontin (OPN) is elevated in human colorectal cancer blood and its expression is correlated with poor prognosis in human colorectal cancer patients. One of the mechanisms underlying OPN function in tumor promotion is inhibition of T cell activation, suggesting that OPN is another immune checkpoint that might compensate for PD-L1 function in suppression of CTL function in colorectal carcinoma. In this study, we determined that OPN expression is elevated in almost all major human cancers and OPN expression is inversely correlated with survival of colon and rectal cancer patients. We have further determined that OPN deficiency leads to increased tumor incidence and tumor growth rate in syngeneic mice, and tumor cells produce OPN to inhibit tumor-specific CTL lytic activity. These findings provide a strong rationale for development of OPN blockade cancer immunotherapy. We have now developed two OPN monoclonal antibodies (100D3 and 103D6) that are effective in enhancing tumor-specific CTL lytic activity in killing colon tumor cells and in suppressing colon tumor growth in vivo. Our data indicate that 100D3 and 103D6 are potentially effective immunotherapeutic agents for colorectal cancer immunotherapy. 

## Data Availability

Publicly available datasets were analyzed in this study. The datasets are described in the method and results section.

## Supplementary Materials

The following are available online at https://www.mdpi.com/2072-6694/13/5/1006/s1, Figure S1. OPN expression level in normal and tumors from human patients, Table S1. Colorectal cancer patient data.

## References

[1] Camus M., Tosolini M., Mlecnik B., Pagès F., Kirilovsky A., Berger A., Costes A., Bindea G., Charoentong P., Bruneval P. (2009). Coordination of Intratumoral Immune Reaction and Human Colorectal Cancer Recurrence. Cancer Res..

[2] Mlecnik B., Van den Eynde M., Bindea G., Church S.E., Vasaturo A., Fredriksen T., Lafontaine L., Haicheur N., Marliot F., Debetancourt D. (2018). Comprehensive Intrametastatic Immune Quantification and Major Impact of Immunoscore on Survival. J. Natl. Cancer Inst..

[3] Le D.T., Uram J.N., Wang H., Bartlett B.R., Kemberling H., Eyring A.D., Skora A.D., Luber B.S., Azad N.S., Laheru D. (2015). PD-1 Blockade in Tumors with Mismatch-Repair Deficiency. N. Engl. J. Med..

[4] Llosa N.J., Cruise M., Tam A., Wicks E.C., Hechenbleikner E.M., Taube J.M., Blosser R.L., Fan H., Wang H., Luber B.S. (2015). The Vigorous Immune Microenvironment of Microsatellite Instable Colon Cancer Is Balanced by Multiple Counter-Inhibitory Checkpoints. Cancer Discov..

[5] Lu C., Yang D., Klement J.D., Oh I.K., Savage N.M., Waller J.L., Colby A.H., Grinstaff M.W., Oberlies N.H., Pearce C.J. (2019). SUV39H1 Represses the Expression of Cytotoxic T-Lymphocyte Effector Genes to Promote Colon Tumor Immune Evasion. Cancer Immunol. Res..

[6] Franzen A., Heinegård D. (1985). Isolation and characterization of two sialoproteins present only in bone calcified matrix. Biochem. J..

[7] Chakraborty G., Jain S., Kundu G.C. (2008). Osteopontin Promotes Vascular Endothelial Growth Factor–Dependent Breast Tumor Growth and Angiogenesis via Autocrine and Paracrine Mechanisms. Cancer Res..

[8] Cheng J., Huo D.-H., Kuang D.-M., Yang J., Zheng L., Zhuang S.-M. (2007). Human Macrophages Promote the Motility and Invasiveness of Osteopontin-Knockdown Tumor Cells. Cancer Res..

[9] Sangaletti S., Tripodo C., Sandri S., Torselli I., Vitali C., Ratti C., Botti L., Burocchi A., Porcasi R., Tomirotti A. (2014). Osteopontin Shapes Immunosuppression in the Metastatic Niche. Cancer Res..

[10] Lim A.M., Rischin D., Fisher R., Cao H., Kwok K., Truong D., McArthur G.A., Young R.J., Giaccia A., Peters L. (2012). Prognostic Significance of Plasma Osteopontin in Patients with Locoregionally Advanced Head and Neck Squamous Cell Carcinoma Treated on TROG 02.02 Phase III Trial. Clin. Cancer Res..

[11] Agrawal D., Chen T., Irby R., Quackenbush J., Chambers A.F., Szabo M., Cantor A., Coppola M., Yeatman T.J. (2002). Osteopontin identified as lead marker of colon cancer progression, using pooled sample expression profiling. J. Natl. Cancer Inst..

[12] Conway C., Mitra A., Jewell R., Randerson-Moor J., Lobo S., Nsengimana J., Edward S., Sanders D.S., Cook M., Powell B. (2009). Gene Expression Profiling of Paraffin-Embedded Primary Melanoma Using the DASL Assay Identifies Increased Osteopontin Expression as Predictive of Reduced Relapse-Free Survival. Clin. Cancer Res..

[13] Donati V., Boldrini L., Dell’Omodarme M., Prati M.C., Faviana P., Camacci T., Lucchi M., Mussi A., Santoro M., Basolo F. (2005). Osteopontin Expression and Prognostic Significance in Non–Small Cell Lung Cancer. Clin. Cancer Res..

[14] Farrokhi V., Chabot J.R., Neubert H., Yang Z. (2018). Assessing the Feasibility of Neutralizing Osteopontin with Various Therapeutic Antibody Modalities. Sci. Rep..

[15] Shang S., Plymoth A., Ge S., Feng Z., Rosen H.R., Sangrajrang S., Hainaut P., Marrero J.A., Beretta L. (2012). Identification of osteopontin as a novel marker for early hepatocellular carcinoma. Hepatology.

[16] Moorman H.R., Poschel D., Klement J.D., Lu C., Redd P.S., Liu K. (2020). Osteopontin: A Key Regulator of Tumor Progression and Immunomodulation. Cancers.

[17] Todaro M., Gaggianesi M., Catalano V., Benfante A., Iovino F., Biffoni M., Apuzzo T., Sperduti I., Volpe S., Cocorullo G. (2014). CD44v6 Is a Marker of Constitutive and Reprogrammed Cancer Stem Cells Driving Colon Cancer Metastasis. Cell Stem Cell.

[18] Huang J., Pan C., Hu H., Zheng S., Ding L. (2012). Osteopontin-Enhanced Hepatic Metastasis of Colorectal Cancer Cells. PLoS ONE.

[19] Chiou J., Chang Y.-C., Tsai H.-F., Lin Y.-F., Huang M.-S., Yang C.-J., Hsiao M. (2019). Follistatin-like Protein 1 Inhibits Lung Cancer Metastasis by Preventing Proteolytic Activation of Osteopontin. Cancer Res..

[20] Sharon Y., Raz Y., Cohen N., Ben-Shmuel A., Schwartz H., Geiger T., Erez N. (2015). Tumor-Derived Osteopontin Reprograms Normal Mammary Fibroblasts to Promote Inflammation and Tumor Growth in Breast Cancer. Cancer Res..

[21] Cantor H., Shinohara M.L. (2009). Regulation of T-helper-cell lineage development by osteopontin: The inside story. Nat. Rev. Immunol..

[22] Patarca R., Freeman G.J., Singh R.P., Wei F.Y., Durfee T., Blattner F., Regnier D.C., A Kozak C., A Mock B., Morse H.C. (1989). Structural and functional studies of the early T lymphocyte activation 1 (Eta-1) gene. Definition of a novel T cell-dependent response associated with genetic resistance to bacterial infection. J. Exp. Med..

[23] Ashkar S., Weber G.F., Panoutsakopoulou V., Sanchirico M.E., Jansson M., Zawaideh S., Rittling S.R., Denhardt D.T., Glimcher M.J., Cantor H. (2000). Eta-1 (Osteopontin): An Early Component of Type-1 (Cell-Mediated) Immunity. Science.

[24] Shinohara M.L., Jansson M., Hwang E.S., Werneck M.B.F., Glimcher L.H., Cantor H. (2005). T-bet-dependent expression of osteopontin contributes to T cell polarization. Proc. Natl. Acad. Sci. USA.

[25] Diao H., Iwabuchi K., Li L., Onoe K., Van Kaer L., Kon S., Saito Y., Morimoto J., Denhardt D.T., Rittling S. (2008). Osteopontin regulates development and function of invariant natural killer T cells. Proc. Natl. Acad. Sci. USA.

[26] Wei J., Marisetty A., Schrand B., Gabrusiewicz K., Hashimoto Y., Ott M., Grami Z., Kong L.-Y., Ling X., Caruso H.G. (2018). Osteopontin mediates glioblastoma-associated macrophage infiltration and is a potential therapeutic target. J. Clin. Investig..

[27] Klement J.D., Paschall A.V., Redd P.S., Ibrahim M.L., Lu C., Yang D., Celis E., Abrams S.I., Ozato K., Liu K. (2018). An osteopontin/CD44 immune checkpoint controls CD8+ T cell activation and tumor immune evasion. J. Clin. Investig..

[28] Katholnig K., Schütz B., Fritsch S.D., Schörghofer D., Linke M., Sukhbaatar N., Matschinger J.M., Unterleuthner D., Hirtl M., Lang M. (2019). Inactivation of mTORC2 in macrophages is a signature of colorectal cancer that promotes tumorigenesis. JCI Insight.

[29] Kim E.-K., Jeon I., Seo H., Park Y.-J., Song B., Lee K.-A., Jang Y., Chung Y., Kang C.-Y. (2014). Tumor-Derived Osteopontin Suppresses Antitumor Immunity by Promoting Extramedullary Myelopoiesis. Cancer Res..

[30] Lin C.-N., Wang C.-J., Chao Y.-J., Lai M.-D., Shan Y.-S. (2015). The significance of the co-existence of osteopontin and tumor-associated macrophages in gastric cancer progression. BMC Cancer.

[31] Kale S.P., Raja R., Thorat D., Soundararajan G., Patil T.V., Kundu G.C. (2013). Osteopontin signaling upregulates cyclooxygenase-2 expression in tumor-associated macrophages leading to enhanced angiogenesis and melanoma growth via α9β1 integrin. Oncogene.

[32] Rao G., Wang H., Li B., Huang L., Xue D., Wang X., Jin H., Wang J., Zhu Y., Lu Y. (2013). Reciprocal Interactions between Tumor-Associated Macrophages and CD44-Positive Cancer Cells via Osteopontin/CD44 Promote Tumorigenicity in Colorectal Cancer. Clin. Cancer Res..

[33] Li Y., Sun B.-S., Pei B., Li C.-G., Zhang Z.-F., Yin Y.-S., Wang C.-L. (2015). Osteopontin-Expressing Macrophages in Non-Small Cell Lung Cancer Predict Survival. Ann. Thorac. Surg..

[34] Zhu Y., Yang J., Xu D., Gao X.-M., Zhang Z., Hsu J.L., Li C.-W., Lim S.-O., Sheng Y.-Y., Zhang Y. (2019). Disruption of tumour-associated macrophage trafficking by the osteopontin-induced colony-stimulating factor-1 signalling sensitises hepatocellular carcinoma to anti-PD-L1 blockade. Gut.

[35] Zhang Y., Du W., Chen Z., Xiang C. (2017). Upregulation of PD-L1 by SPP1 mediates macrophage polarization and facilitates immune escape in lung adenocarcinoma. Exp. Cell Res..

[36] Shurin M.R. (2018). Osteopontin controls immunosuppression in the tumor microenvironment. J. Clin. Investig..

[37] Zhang L., Li Z., Skrzypczynska K.M., Fang Q., Zhang W., O’Brien S.A., He Y., Wang L., Zhang Q., Kim A. (2020). Single-Cell Analyses Inform Mechanisms of Myeloid-Targeted Therapies in Colon Cancer. Cell.

[38] Azizi E., Carr A.J., Plitas G., Cornish A.E., Konopacki C., Prabhakaran S., Nainys J., Wu K., Kiseliovas V., Setty M. (2018). Single-Cell Map of Diverse Immune Phenotypes in the Breast Tumor Microenvironment. Cell.

[39] Shankaran V., Ikeda H., Bruce A.T., White J.M., Swanson P.E., Old L.J., Schreiber R.D. (2001). IFNgamma and lymphocytes prevent primary tumour development and shape tumour immunogenicity. Nature.

[40] Qin Z., Kim H.-J., Hemme J., Blankenstein T. (2002). Inhibition of Methylcholanthrene-induced Carcinogenesis by an Interferon γ Receptor–dependent Foreign Body Reaction. J. Exp. Med..

[41] Zimmerman M., Hu X., Liu K. (2010). Experimental Metastasis and CTL Adoptive Transfer Immunotherapy Mouse Model. J. Vis. Exp..

[42] Aran D., Looney A.P., Liu L., Wu E., Fong V., Hsu A., Chak S., Naikawadi R.P., Wolters P.J., Abate A.R. (2019). Reference-based analysis of lung single-cell sequencing reveals a transitional profibrotic macrophage. Nat. Immunol..

[43] Wu C.-Y., Wu M.-S., Chiang E.-P., Chen Y.-J., Chi N.-H., Chen G.-H., Lin J.-T. (2007). Elevated plasma osteopontin associated with gastric cancer development, invasion and survival. Gut.

[44] Coppola D., Szabo M., Boulware D., Muraca P., Alsarraj M., Chambers A.F., Yeatman T.J. (2004). Correlation of Osteopontin Protein Expression and Pathological Stage across a Wide Variety of Tumor Histologies. Clin. Cancer Res..

[45] Zhang B., Dai J., Wang H., Wei H., Zhao J., Guo Y., Fan K. (2014). Anti-osteopontin monoclonal antibody prevents ovariectomy-induced osteoporosis in mice by promotion of osteoclast apoptosis. Biochem. Biophys. Res. Commun..

[46] Yamamoto N., Nakashima T., Torikai M., Naruse T., Morimoto J., Kon S., Sakai F., Uede T. (2007). Successful treatment of collagen-induced arthritis in non-human primates by chimeric anti-osteopontin antibody. Int. Immunopharmacol..

[47] Fan K., Dai J., Wang H., Wei H., Cao Z., Hou S., Qian W., Wang H., Li B., Zhao J. (2008). Treatment of collagen-induced arthritis with an anti-osteopontin monoclonal antibody through promotion of apoptosis of both murine and human activated T cells. Arthritis Rheum..

[48] Zhao F., Zhang Y., Wang H., Jin M., He S., Shi Y., Guo Y., Zhang Y. (2011). Blockade of osteopontin reduces alloreactive CD8+ T cell–mediated graft-versus-host disease. Blood.

[49] Shojaei F., Scott N., Kang X., Lappin P.B., A Fitzgerald A., Karlicek S., Simmons B.H., Wu A., Lee J.H., Bergqvist S. (2012). Osteopontin induces growth of metastatic tumors in a preclinical model of non-small lung cancer. J. Exp. Clin. Cancer Res..

[50] Maeda N., Ohashi T., Chagan-Yasutan H., Hattori T., Takahashi Y., Harigae H., Hasegawa H., Yamada Y., Fujii M., Maenaka K. (2015). Osteopontin-integrin interaction as a novel molecular target for antibody-mediated immunotherapy in adult T-cell leukemia. Retrovirology.

[51] Chiodoni C., Sangaletti S., Tripodo C., Colombo M.P. (2015). The ins and outs of osteopontin. OncoImmunology.

[52] Masugi Y., Nishihara R., Mingyang S., Mima K., Da Silva A., Shi Y., Inamura K., Cao Y., Song M., Nowak J.A. (2017). Tumour CD274 (PD-L1) expression and T cells in colorectal cancer. Gut.

[53] Marisa L., Svrcek M., Collura A., Becht E., Cervera P., Wanherdrick K., Buhard O., Goloudina A., Jonchère V., Selves J. (2018). The Balance Between Cytotoxic T-cell Lymphocytes and Immune Checkpoint Expression in the Prognosis of Colon Tumors. J. Natl. Cancer Inst..

[54] He B., Mirza M., Weber G.F. (2005). An osteopontin splice variant induces anchorage independence in human breast cancer cells. Oncogene.

[55] Briones-Orta M.A., Avendaño-Vázquez S.E., Aparicio-Bautista D.I., Coombes J.D., Weber G.F., Syn W.-K. (2017). Osteopontin splice variants and polymorphisms in cancer progression and prognosis. Biochim. Et Biophys. Acta (BBA) Bioenerg..

[56] Lin Y.-H., Yang-Yen H.-F. (2001). The Osteopontin-CD44 Survival Signal Involves Activation of the Phosphatidylinositol 3-Kinase/Akt Signaling Pathway. J. Biol. Chem..

[57] Weber G.F., Ashkar S., Glimcher M.J., Cantor H. (1996). Receptor-Ligand Interaction Between CD44 and Osteopontin (Eta-1). Science.

