# Supplementary material for: Osteopontin Blockade Immunotherapy Increases Cytotoxic T Lymphocyte Lytic Activity and Suppresses Colon Tumor Progression
Source: Cancers (Basel). 2021 Feb 28;13(5):1006. doi: 10.3390/cancers13051006 (PMC7957528; doi:10.3390/cancers13051006)
Supplement: Supplementary file 1 [file cancers-13-01006-s001.pdf]

Supplementary Materials: Osteopontin Blockade Immunotherapy Increases Cytotoxic T Lymphocyte Lytic Activity and Suppresses Colon Tumor Progression

John D. Klement, Dakota B. Poschel, Chunwan Lu, Alyssa D. Merting, Dafeng Yang, Priscilla S. Redd and Kebin Liu

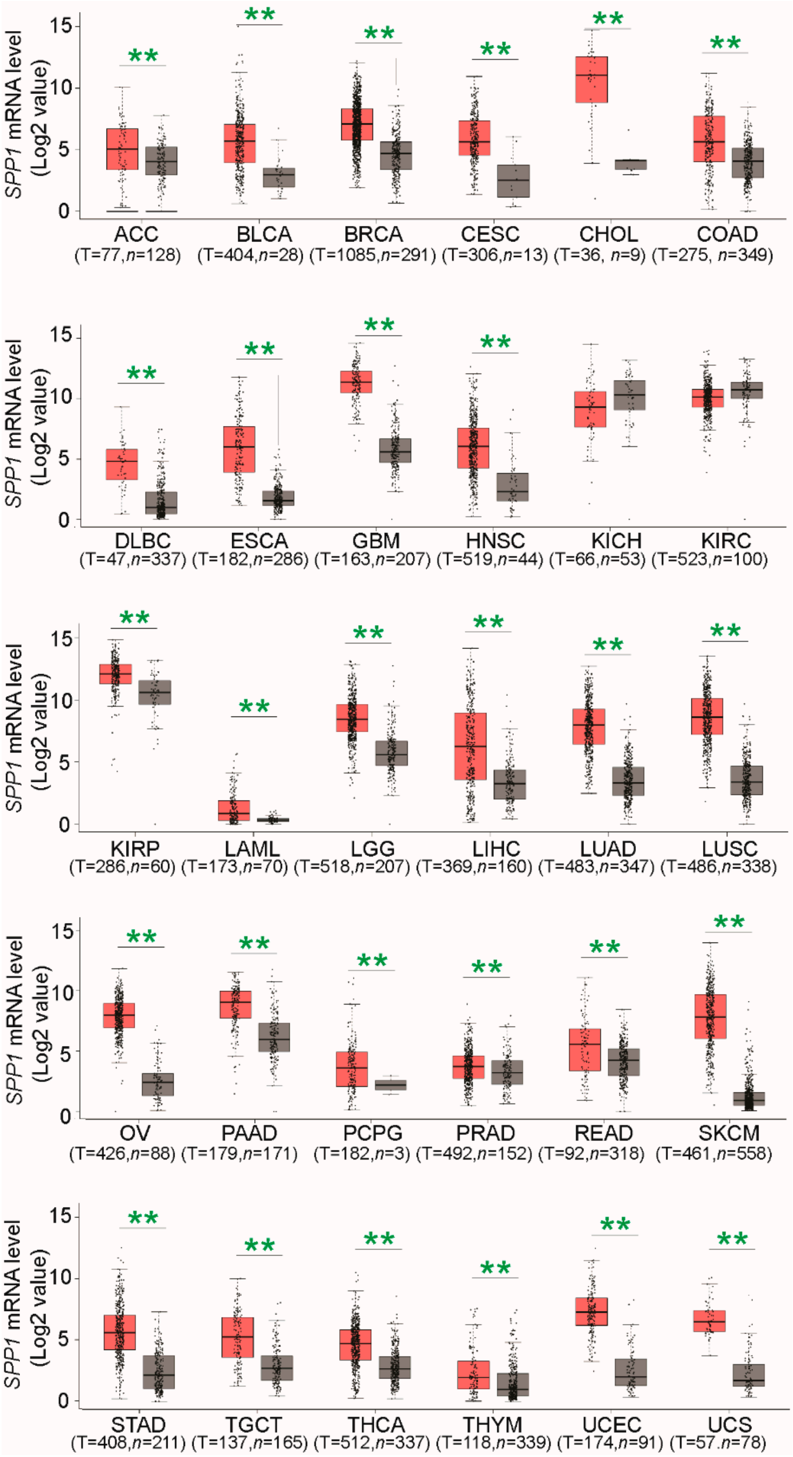

Figure S1. OPN expression level in normal and tumors from human patients. OPN mRNA expression datasets in the indicated human tumors and normal tissues were extracted from GEPIA (Gene Expression Profiling Interactive Analysis) database. OPN expression level between tumor

and normal tissue in each tumor type was then plotted and analyzed. ** $p < 0.01$. Tumor types: ACC: Adrenocortical carcinoma; BLCA: Bladder Urothelial Carcinoma; BRCA: Breast invasive carcinoma; CESC: Cervical squamous cell carcinoma and endocervical adenocarcinoma; CHOL: Cholangio carcinoma; COAD: Colon adenocarcinoma; DLBC: Lymphoid Neoplasm Diffuse Large B-cell Lymphoma; ESCA: Esophageal carcinoma; GBM: Glioblastoma multiforme; HNSC: Head and Neck squamous cell carcinoma; KICH: Kidney Chromophobe; KIRC: Kidney renal clear cell carcinoma; KIRP: Kidney renal papillary cell carcinoma; LAML: Acute Myeloid Leukemia; LGG: Brain Lower Grade Glioma; LIHC: Liver hepatocellular carcinoma; LUAD: Lung adenocarcinoma; LUSC: Lung squamous cell carcinoma; OV: Ovarian serous cystadenocarcinoma; PAAD: Pancreatic adenocarcinoma; PCPG: Pheochromocytoma and Paraganglioma; PRAD: Prostate adenocarcinoma; READ: Rectum adenocarcinoma; SKCM: Skin Cutaneous Melanoma; STAD: Stomach adenocarcinoma; TGCT: Testicular Germ Cell Tumors; THCA: Thyroid carcinoma; THYM: Thymoma; UCEC: Uterine Corpus Endometrial Carcinoma; UCS: Uterine Carcinosarcoma.

Table S1. Colorectal cancer patient data.

Patient	Gender	Age	Race	Type	Description	Pathologic Stage	Tumor	Treatment:
Patient 1	Male	73	Caucasian	colon, transverse and liver	invasive moderately differentiated adenocarcinoma, with liver metastasis	N/A	primary	Chemotherapy
Patient 2	Male	55	Caucasian	rectosigmoid junction	invasive moderately to poorly differentiated adenocarcinoma with liver metastasis	N/A	primary	Chemotherapy and radiation
Patient 3	Male	57	Caucasian	colon, ascending and liver	invasive moderately differentiated adenocarcinoma, liver metastasis	Stage IV (T3N2M1)	primary	Chemotherapy and radiation
Patient 4	Male	75	Caucasian	colon, ascending and liver	invasive adenocarcinoma of the colon, grade 1. Liver metastasis	pT3b, pN2, pM1	primary	Chemotherapy adjuvant
Patient 5	Male	64	Black	colon, cecum, liver	metastatic adenocarcinoma, liver metastasis	pTa, p1, pM1	primary	Radiation therapy and chemotherapy

N/A not available

Copyright: © 2021 by the authors. Licensee MDPI, Basel, Switzerland. This article is an open access article distributed under the terms and conditions of the Creative Commons Attribution (CC BY) license (http://creativecommons.org/licenses/by/4.0/).
